# EVI5 is an oncogene that regulates the proliferation and metastasis of NSCLC cells

**DOI:** 10.1186/s13046-020-01585-z

**Published:** 2020-05-11

**Authors:** Tingting Cai, Jieqi Zhou, Yuanyuan Zeng, Wenwen Du, Yang Zhang, Ting Liu, Yulong Fu, Jian-an Huang, Qian Qian, Jianjie Zhu, Chunhua Ling, Zeyi Liu

**Affiliations:** 1 Department of Respiratory Medicine, the First Affiliated Hospital of Soochow University, Suzhou, 215006 China; 2 Suzhou Key Laboratory for Respiratory Diseases, Suzhou, 215006 China; 3 Institute of Respiratory Diseases, Soochow University, Suzhou, 215006 China; 4 Department of Medicine, Division of Allergy and Clinical Immunology, National Jewish Health, Denver, CO 80206 USA

**Keywords:** EVI5, Emi1, TGF-β receptor II, TGF-β receptor I, miR-486-5p, NSCLC

## Abstract

**Background:**

The Ecotropic viral integration site 5 (EVI5), an important protein in regulating cell cycle, cytokinesis and cellular membrane traffic, functions as a stabilizing factor maintaining anaphase-promoting complex/cyclosome (APC/C) inhibitor Emi1 in S/G2 phase. However, the mechanism by which EVI5 promotes malignant transformation of non-small cell lung cancer (NSCLC) remains unknown. In the present study, we addressed the role of EVI5 in NSCLC by regulating tumor growth, migration and invasion.

**Methods:**

The expression levels of EVI5 and miR-486-5p in NSCLC tissues and cells were measured by real-time PCR. Meanwhile, EVI5 and its associated protein expression were analyzed by western blot and co-immunoprecipitation assay. Flow cytometry was performed to determine cell proliferation and apoptosis. CCK-8 and clonogenic assays were used to analyze cell viability. Wound healing, transwell migration and matrigel invasion assays were utilized to assess the motility of tumor cells. To investigate the role of EVI5 in vivo, lung carcinoma xenograft mouse model was applied..

**Results:**

EVI5 was upregulated in NSCLC tissues and cell lines when compared with that in normal tissues and cell line. Knockdown of EVI5 in vitro inhibited tumor cell proliferation, migration and invasion in NSCLC cells. Further, inoculation of EVI5-deficient tumor cells into nude mice suppressed tumor proliferation and metastasis compared to control mice inoculated with unmanipulated tumor cells. These data indicated that EVI5 promote the proliferation of NSCLC cells which was consistent with our previous results. Additionally, we showed that EVI5 was directly regulated by miR-486-5p, and miR-486-5p-EVI5 axis affected the NSCLC migration and invasion through TGF-β/Smad signaling pathway by interacting with TGF-β receptor II and TGF-β receptor I.

**Conclusions:**

Based on these results, we demonstrated a new post-transcriptional mechanism of EVI5 regulation via miR-486-5p and the protumoral function of EVI5 in NSCLC by interacting with Emi1 and/or TGF-β receptors, which provides a new insight into the targeted therapy of NSCLC.

## Background

Lung cancer is the primary cause of cancer-related death worldwide; NSCLC is the main type, accounting for approximately 85% of lung cancers [1–3]. Despite the in-depth approaches to considerable innovations in targeted therapy, the survival of NSCLC patients is still not ideal, and the 5-year survival rate is less than 15% [4]. Thus, the need to identify potential molecular targets for the treatment of NSCLC is urgent. In this paper, we demonstrated that EVI5 functions as an oncogene in the pathogenesis of NSCLC.

EVI5 belongs to a small subfamily of Tre-2/Bub2/Cdc16 (TBC) domain-containing proteins [5], which play enigmatically divergent roles as modulators of cell cycle progression, cytokinesis, and cellular membrane trafficking [6, 7]. EVI5 contains a centrosomal targeting domain with homology to the structural maintenance of chromosomes (SMC) family of ATPases in the C-terminal half and a TBC domain in the N-terminal region [8]; TBC domains are commonly associated with a functional class of proteins that act as GTPase-activating proteins (GAPs) for the Rab family GTPases and regulate membrane trafficking [9].

EVI5 functions as a stabilizing factor which maintains Emi1 level in S/G2 phase of cell cycle, which resulted in the accumulation of Cyclins [10]. Furthermore, a study showed that the expression of Emi1 is upregulated in several solid tumors, including NSCLC [11]. Thus, we hypothesize that EVI5 could also promote the cell cycle of NSCLC by combining with Emi1 to induce Cyclins accumulation.

Noteworthy, tumor metastatic invasiveness is linked with the increased migration capacity of epithelial cells. This process is known as the epithelial-mesenchymal transition (EMT) [12]. EMT is essential for morphogenesis during embryonic development and early tumour transformation into invasive malignancies [13–15]. In particular, accumulating evidence indicates that TGF-β/Smad signaling is a potent inducer of EMT in various cancers, including NSCLC [16, 17]. TGF-β/Smad signaling pathway can regulate cell proliferation, migration, invasion, and other functions through a complex pathway network comprising multiple pathways [18, 19]. EVI5 could promote collective cell migration through its Rab-GAP activity [20], the mechanism by which it regulates tumor metastasis remains unclear. Data extracted from GEPIA2 database (http://gepia.cancer-pku.cn/) showed that the level of EVI5 is positively correlated with that of TGF-β receptors in NSCLC, especially TGF-β receptor II, which is an initiator of TGF-β/Smad signaling [21]. Thus, we sought to investigate whether EVI5 could interact with TGF-β/Smad signaling pathway, which may promote the EMT progression of NSCLC, which would be a particularly important clinical significance.

In present study, the interaction between Emi1 and EVI5 suggested that EVI5 does play a protumoral role in NSCLC, in which the binding of EVI5 and Emi1 is the key to the accumulation of Cyclins. More important, we found a significant phenotype that EVI5 influence the migration and invasion of NSCLC by interacting with TGF-β receptors to activate the downstream TGF-β/Smad signaling pathway. In addition, we found that miR-486-5p represses EVI5 expression, and the low expression level of miR-486-5p may be one of the reasons for the elevated expression of EVI5 in NSCLC. Here, we aimed to evaluate the role of EVI5 in the tumorigenesis of NSCLC, and to explore the possible role of miR-486-5p in EVI5 dysregulation in lung carcinogenesis.

## Methods

### Tissue samples

Paired NSCLC and adjacent noncancerous lung tissue samples (60 of each) were collected with the informed consent of the patients from the First Affiliated Hospital of Soochow University between 2015 and 2018. The patients had been diagnosed with NSCLC based on their histological and pathological characteristics according to the Revised International System for Staging Lung Cancer. No patient had received chemotherapy or radiotherapy before tissue sampling. The tissue samples were snap frozen and stored in a cryofreezer at − 80 °C. This study was approved by the Ethics Committee of the First Affiliated Hospital of Soochow University.

### Cell lines and cultures

The human NSCLC cell lines A549, H226, H1299, H1650, SPC-A1 and H460 and the human immortalized normal epithelial cell line 16HBE were obtained from the Cell Bank of the Chinese Academy of Sciences (Shanghai, China). Cells were cultured in Roswell Park Memorial Institute (RPMI) 1640 medium containing 10% foetal bovine serum (FBS) (Gibco, Carlsbad, CA, USA) and L-glutamine (Invitrogen, Carlsbad, CA, USA) at 37 °C in a humidified atmosphere containing 5% CO_2_.

### RNA interference

Two pre-designed small interfering RNA (siRNA) sequences targeting different coding regions of EVI5 were directly synthesized (GenePharma). The target sequences of the siRNAs were as follows: siRNA-EVI5–1: 5′-GAG UCU CAG UGU GCA UUA ATT-3′; siRNA-EVI5–2: 5′-GGA CUC CUU ACU CAA UUA ATT-3′; and siRNA-Emi1: 5′-GCA CUA GAG ACC AGU AGA CTT-3′. Scrambled siRNA was used as a negative control. Cells were transiently transfected with 50 nM siRNA sequences using Lipofectamine 3000 (Invitrogen, Waltham, MA, USA). After 72 h of transfection, cells were harvested for further experiments.

### Establishment of stable EVI5-overexpressing cell lines

To generate NSCLC cells in which EVI5 was stably overexpressed, a 2493-bp fragment of the EVI5 coding sequence was synthesized (Genewiz, Suzhou, China) and subcloned into the PLVX-IRES-Neo vector (PLVX) using the endonucleases EcoRI and XbaI for expression in a Lenti-X lentiviral expression system (Clontech, Mountain View, CA, USA). Empty vector was used as a negative control. HEK293T cells were cultured in Dulbecco’s modified Eagle’s medium containing 10% FBS at 37 °C in a humidified 5% CO_2_ incubator for 48 h. The EVI5 expression construct was co-transfected with packaging plasmids into HEK293T cells using Lipofectamine 3000. After incubation, the packaged lentiviruses were collected and used to infect A549 and H226 cells. After 48 h, stable cells were selected with 400 μg/ml G418 (Amresco, Solon, OH, USA).

### Establishment of stable EVI5-knockout cell lines

To establish stable cell lines with silenced EVI5 expression, guide RNA (gRNA) sequences were synthesized (Genewiz). The target sequences of the EVI5-gRNA were as follows: Forward: 5′-CACCGAAAGGCAGCAGTCATTTTGT-3′ and Reverse: 5′-AAACACAAAATGACTGCTGCCTTTC-3′. Then, we subcloned the EVI5-gRNA into the lentiviral vector Lenti-CRISPR v2 (Cas-9, GenePharma, Shanghai, China) digested with BsmBI after phosphorylation and annealing. The correctness of the Lenti-CRISPR-sgEVI5 (EVI5-KO) plasmid was confirmed by sequencing. Empty vector was used as a negative control. Then, the EVI5 silencing construct or the negative control was co-transfected with packaging plasmids into HEK293T cells using Lipofectamine 3000 (Invitrogen). After incubation, the packaged lentiviruses were collected and used to infect A549 and H226 cells. After 48 h, stable cells were selected with 0.4 μg/ml or 2 μg/ml puromycin (Sigma-Aldrich, St. Louis, MO, USA).

### RNA extraction, cDNA synthesis, and quantitative real-time PCR (qRT-PCR)

Total RNA was extracted from cells by the addition of 1 ml of RNAiso Plus (Takara, Osaka, Japan) according to the manufacturer’s protocol. The RNA concentration was measured using a NanoDrop 2000 (Thermo Fisher Scientific, Waltham, MA, USA). cDNA synthesis was carried out with M-MLV reverse transcriptase (Takara). The primers used for reverse transcription and amplification of miR-486-5p and U6 were designed and synthesized by Guangzhou RiboBioCorp (Guangzhou, China). The primers for EVI5 and β-actin used for qRT-PCR analysis were as follows: EVI5, Forward: 5′-GCATCATCCTGGTTTCTGAC-3′ and Reverse: 5′-AGCTTGTCTGGG ACACCATC-3′; and β-actin, Forward: 5′-CACAGAGCCTCGCCTTTGCC-3′ and Reverse: 5′-ACCCATGCCCACCATCACG-3′. The primers specific for U6 were purchased from RiboBioCo., Ltd. (Guangzhou, China). qRT-PCR was performed using SYBR Premix ExTaq™ (Takara) according to the manufacturer’s instructions with an ABI Step One Plus Real-Time PCR system (Applied Biosystems). The PCR program was as follows: 95 °C for 10 min, followed by 40 cycles at 95 °C for 15 s and 60 °C for 1 min. The expression values of EVI5 mRNA and miR-486-5p were normalized to those of the internal controls β-actin and U6, respectively. Relative expression was calculated using the ^ΔΔ^Ct method [22].

### Western blotting assay

Western blot analysis was performed as previously described by us [23]. The following antibodies were used in the analysis: anti-EVI5 (Millipore, Billerica, MA, USA); anti-Emi1 and anti-TGF-β receptor II (Santa Cruz, CA, USA); anti-CyclinA2 (Proteintech, IL, USA); anti-pAkt (Ser473), anti-Akt, anti-Erk1/2, anti-pErk (Thr202/Tyr204), anti-CyclinD1, anti-MMP2, anti-p-Smad3, anti-Snail and anti-β-actin (Cell Signaling Technology, Danvers, MA, USA); anti-TGF-β receptor I (Abcam, London, UK); anti-N-cadherin and anti-Vimentin (BD Biosciences, USA); Anti-mouse and anti-rabbit secondary antibodies (Cell Signaling Technology, Danvers, MA, USA).

### Co-immunoprecipitation (co-ip) assay

NSCLC cells were cultured in a 100 mm plate to 95–100% confluence. Then, the cells in each dish were washed twice with cold phosphate-buffered saline (PBS), collected by scraping, and lysed with 1 ml of modified RIPA buffer (Cell Signaling Technology, Danvers, MA, USA) containing protease and phosphatase inhibitor cocktail (Sigma-Aldrich, St. Louis, MO, USA) for 30 min. Cell lysates were collected by centrifugation at 10,000×g at 4 °C for 30 min. Clear lysates were pre-cleared by the addition of 50 μl of protein G bead slurry and incubated at 4 °C overnight with rotation. Supernatants were transferred to a new Eppendorf tube and incubated with 1 μg of rabbit anti-EVI5 antibody (Abcam, ab70790) with rotation overnight in a cold room; this step was followed by an additional incubation for 3–4 h with protein G beads. The beads were washed three times with RIPA buffer and then boiled in 2× SDS protein loading buffer for 5 min. Samples (20 μl) were loaded on SDS-PAGE gels for western blot analysis.

### Cell viability assay

Cell proliferation was examined using a Cell Counting Kit-8 (CCK-8) (Beyotime, Shanghai, China). Tumour cells were seeded in 96-well plates at a density of 3 × 10^3^ cells per well and further grown under normal culture conditions for 24, 48 and 72 h. Cell viability was measured according to the manufacturer’s instructions at several time points (24, 48 and 72 h). We also assessed cell proliferation using a clonogenic assay. Briefly, tumour cells were diluted in complete culture medium, and 3000 cells were reseeded in a 60-mm plate. After incubation for 7–10 days, depending on the cell growth rate, colonies formed by at least 50 cells were stained with Giemsa and counted. Each experiment was performed in triplicate.

### Wound healing assay

A wound healing assay was performed as described previously [23]. Briefly, tumour cells were seeded into 6-well cell culture plates after 24 h of transfection and cultured in a monolayer to 70–80% confluence. The monolayer was gently and slowly scratched using a fresh 10-μl pipette tip across the centre of the well, aiming for a resulting gap distance equal to the outer diameter of the end of the tip. Another scratch was made perpendicular to the first to create a cross in each well. Detached cells were then removed by two gentle washes with 1 × PBS. The well was replenished with fresh medium, and cells were cultured for an additional 24 h. Cells were observed and imaged under a microscope (CKX41, Olympus) at the same magnification and settings. The width of the gap was evaluated quantitatively using Photoshop.

### Migration and invasion assays

Transwell migration and invasion assays were performed as described previously [23]. For the migration assay, 3 × 10^4^ tumour cells in medium containing 1% FBS were seeded onto the upper chamber of a transwell insert, and 800 μl of medium containing 10% FBS was added to the lower chamber. For the invasion assay, 5 × 10^4^ tumour cells in medium containing 1% FBS were seeded onto the upper chamber of a transwell insert coated with Matrigel matrix (BD Science, Sparks, MD, USA), and 800 μl of medium containing 10% FBS was added to the lower chamber. At 6 h later, if necessary, TGF-β1 (5 ng/ul) was added to the lower chambers, and the plates were incubated at 37 °C for 24 h. After 24 h of incubation, the cells that had migrated onto the lower surface of the chamber were fixed with 100% methanol and stained with 1% crystal violet. Finally, the cells were counted in at least three random fields under a light microscope.

### Cell cycle analysis

According to the protocol of the Cell Cycle Analysis Kit (Beyotime, Shanghai, China), cells were cultured in 6-well plates and transfected with negative control miRNA (miR-NC), miR-486-5p, si-NC or si-EVI5 for 48 h. Cells were then harvested, washed with cold PBS, fixed with 70% ethanol at 4 °C for 24 h, washed with cold PBS again and stained with a propidium iodide (PI)/RNase A mixture. Next, cells were incubated in the dark at 37 °C for 30 min and analysed using a fluorescence-activated cell sorting (FACS) Calibur system (Beckman Coulter, Brea, CA, USA).

### Cell apoptosis analysis

According to the protocol of the Annexin V-FITC Apoptosis Detection Kit (Beyotime, Shanghai, China), cells were transfected with miR-NC, miR-486-5p, si-NC or si-EVI5. After 48 h, cells were harvested, washed with cold PBS, and resuspended in binding buffer containing Annexin V/FITC and PI (Beyotime). Stained cells were then detected using the FACS Calibur system (Beckman Coulter).

### Xenografts

BALB/c athymic nude mice (female, 4–6 weeks old, weighing 16–20 g) were purchased from the Experimental Animal Center of Soochow University and bred under pathogen-free conditions. All animal experiments were carried out in accordance with the Guide for the Care and Use of Experimental Animals of the Experimental Animal Center of Soochow University. Two million stable EVI5 cells (A549-Cas-9 cells and A549-EVI5-KO cells) were suspended in 150 μl of FBS-free medium and subcutaneously injected into the nude mice, which were randomly divided into two groups (8 mice per group). Tumour growth was analysed by measuring the tumour length (L) and width (W) and calculating the volume (V) with the formula V = LW^2^/2.

### Statistical analysis

All numerical data are presented as the mean ± SD. Statistical analysis was performed with an unpaired t test (two-tailed). A paired t test (two-tailed) was performed to determine the significance of the data from patient samples. Differences for which P was < 0.05 were considered significant. Statistical analyses were conducted using GraphPad Prism 7 software (GraphPad, San Diego, CA, USA).

## Results

### High incidence of EVI5 overexpression in NSCLC tissues and cell lines

**Fig. 1:**
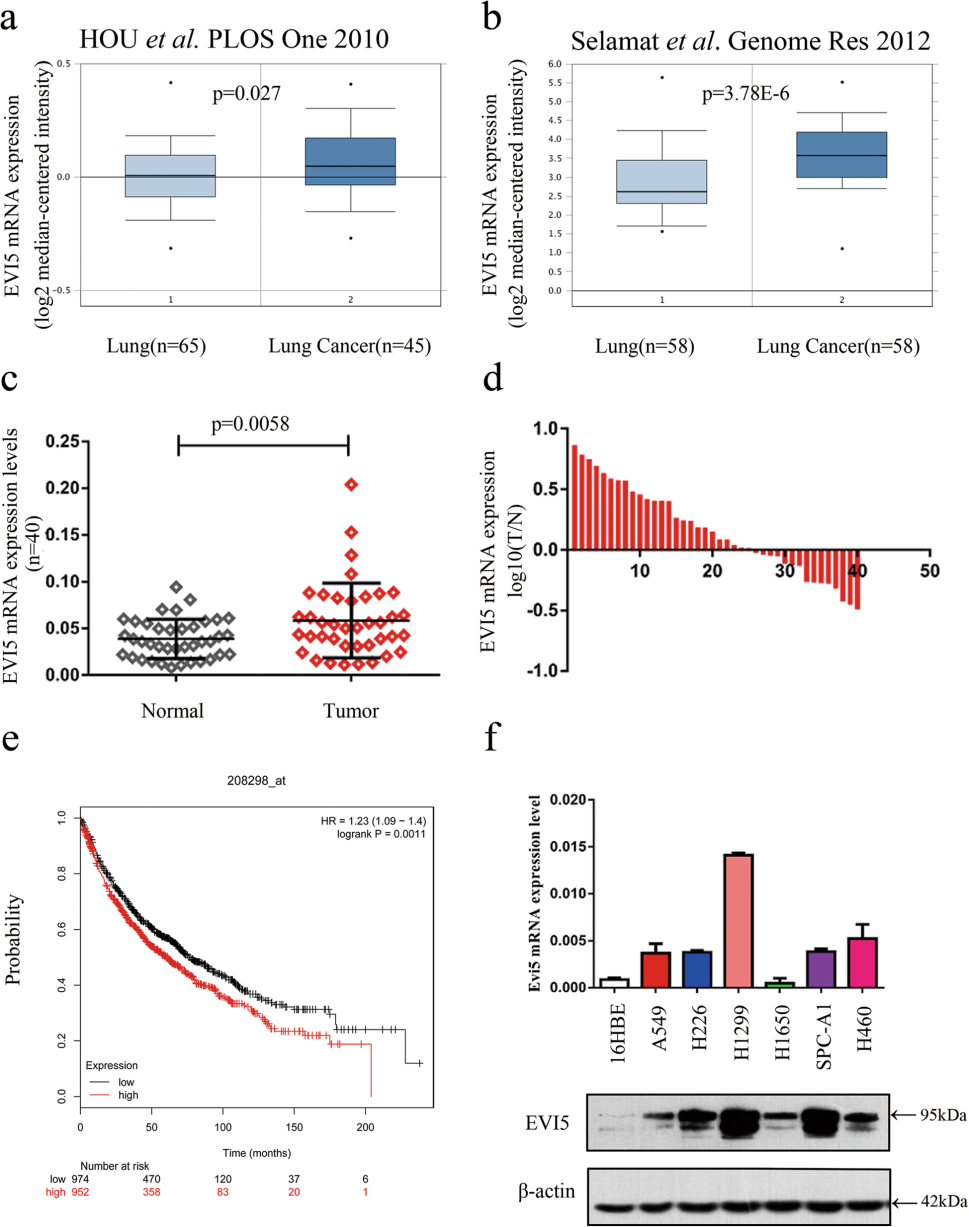
EVI5 is upregulated in NSCLC tissues and cell lines. **a-b** Data obtained from several study groups deposited in the Oncomine database (http://www.oncomine.org) were analysed to compare the differences in EVI5 expression between lung cancer and normal lung tissue. **c** EVI5 mRNA levels in 40 NSCLC tissues and paired oncancerous lung tissues. **d** Relative mRNA expression levels of EVI5 in 40 paired NSCLC tissues. The Y axis indicates the log10 transformed fold change in the T/N protein expression ratios of EVI5. The number of each specimen is indicated below the X axis. **e** Effect of the EVI5 mRNA expression level on overall survival in 1926 NSCLC patients. Kaplan–Meier plots were generated using Kaplan–Meier Plotter (http://www.kmplot.com). **f** Total RNA and protein were extracted from several cell lines, and the expression of EVI5 at the mRNA and protein levels was measured by qRT-PCR and western blotting, respectively. β-actin was used as the internal control. Bars represent mean ± SD from three independent experiments. Significant differences compared with the control: * *P* < 0.05; **P < 0.01; ***P < 0.001

Data extracted from Oncomine (http://www.oncomine.org) showed that EVI5 mRNA expression was significantly upregulated in lung cancer compared to that of normal lung tissues (Fig. 1a, b, *P* < 0.05). We then verified the expression of EVI5 mRNA in 40 paired NSCLC tissues and adjacent noncancerous lung tissues, result showed that the EVI5 mRNA levels was significantly higher in NSCLC tissues than that in adjacent noncancerous lung tissues (Fig. 1c, *P* < 0.01). As illustrated in Fig. 1d, there were 24 (60%) cases of NSCLC with higher level of EVI5 mRNA expression among the total of 40 paired NSCLC tissues. However, there was no correlation between the mRNA expression of EVI5 and clinicopathological parameters (Additional file 1: Table S1); More important, dataset extracted from Kaplan-Meier Plotter (http://www.kmplot.com) indicated that mRNA expression level of EVI5 was significantly associated with poor survival of patients with NSCLC (Fig. 1e, *P* < 0.01). In addition, we detected EVI5 mRNA and protein expression levels in 6 NSCLC cell lines along with a normal bronchial epithelial cell line, 16HBE. The results showed that the EVI5 levels in the NSCLC cell lines were significantly higher than that in the normal cell sample (Fig. 1f). Collectively, our data showed that EVI5 is upregulated in NSCLC tissues and cell lines.

### Knockdown of EVI5 inhibits cell proliferation, migration and invasion in vitro

**Fig. 2:**
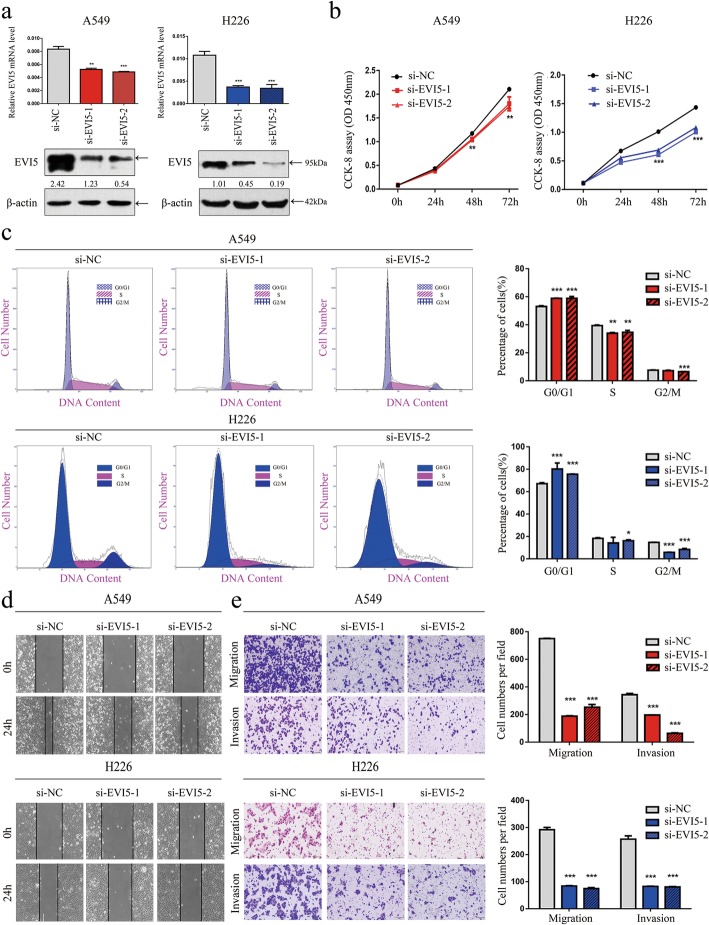
Knockdown of EVI5 inhibits cell proliferation, migration and invasion in vitro. **a** EVI5 mRNA and protein levels in EVI5-knockdown NSCLC cells. **b** CCK-8 assay of cell viability in A549 and H226 cells (si-EVI5 compared with si-NC). **c** Flow cytometry assay of A549 and H226 cells (si-EVI5 compared with si-NC). Cells were harvested 72 h after transfection and stained with PI. The percentage of cells in each cell cycle phase is shown in the inset of each panel. **d** Wound healing assay was performed to evaluate cell migration in A549 and H226 cells (si-EVI5 compared with si-NC). **e** Representative images of the transwell assay results for cell migration and invasion in A549 and H226 cells (si-EVI5 compared with si-NC). β-actin was used as the internal control. Bars represent mean ± SD from three independent experiments. Significant differences compared with the control: * *P* < 0.05; **P < 0.01; ***P < 0.001

The expression level of EVI5 mRNA and protein was significantly reduced after transfection of A549 and H226 cells with two siRNA against EVI5 (Fig. 2a). The CCK-8 assay showed that cell proliferation was significantly inhibited in cells with knockdown of EVI5 compared with the control cells at 24 h, 48 h, and 72 h (Fig. 2b). We also confirmed these findings by performing a clonogenic assay (Additional file 2: Figure S1a). Further, the proportion of cells in the G0/G1 phase was significantly higher and the proportion of cells in the S phase was significantly lower in the EVI5-knockdown cells than that in the control cells (Fig. 2c, *P* < 0.01). The flow cytometry results indicated that NSCLC cells transfected with EVI5 knockdown siRNA exhibited an increase in apoptosis (Additional file 2: Figure S1b). In the wound healing assay, the si-EVI5-transfected NSCLC cells migrated towards to the scratch more slowly than did the control cells (Fig. 2d). Transwell assay of the NSCLC cells lines further indicated that loss of EVI5 considerably suppressed the migration and invasion abilities of NSCLC cells (Fig. 2e). These results indicated that EVI5 can inhibit cell proliferation in NSCLC cells via its effects on cell cycle and apoptosis.

### Increased cell proliferation, migration and invasion abilities of NSCLC cells in vitro by EVI5 overexpression

To further investigate the function of EVI5 in NSCLC cells, we established stable lines with overexpression of EVI5, this was confirmed by the increased level of EVI5 mRNA and protein expression in the NSCLC cells compared to that in vector transfected cells (Additional file 3: Figure S2a). CCK-8 and clonogenic assays showed that the proliferation of cells with overexpressed EVI5 was significantly promoted when compared with that in control cells (Additional file 3: Figure S2b, c). Moreover, transwell assay showed that overexpression of EVI5 enhanced the migration and invasion of NSCLC cells (Additional file 3: Figure S2d). Collectively, these data strongly indicated that the overexpression of EVI5 promotes the proliferation, migration and invasion of NSCLC.

### Inhibition of tumor growth and lung metastasis in vivo by EVI5 knockout

**Fig. 3:**
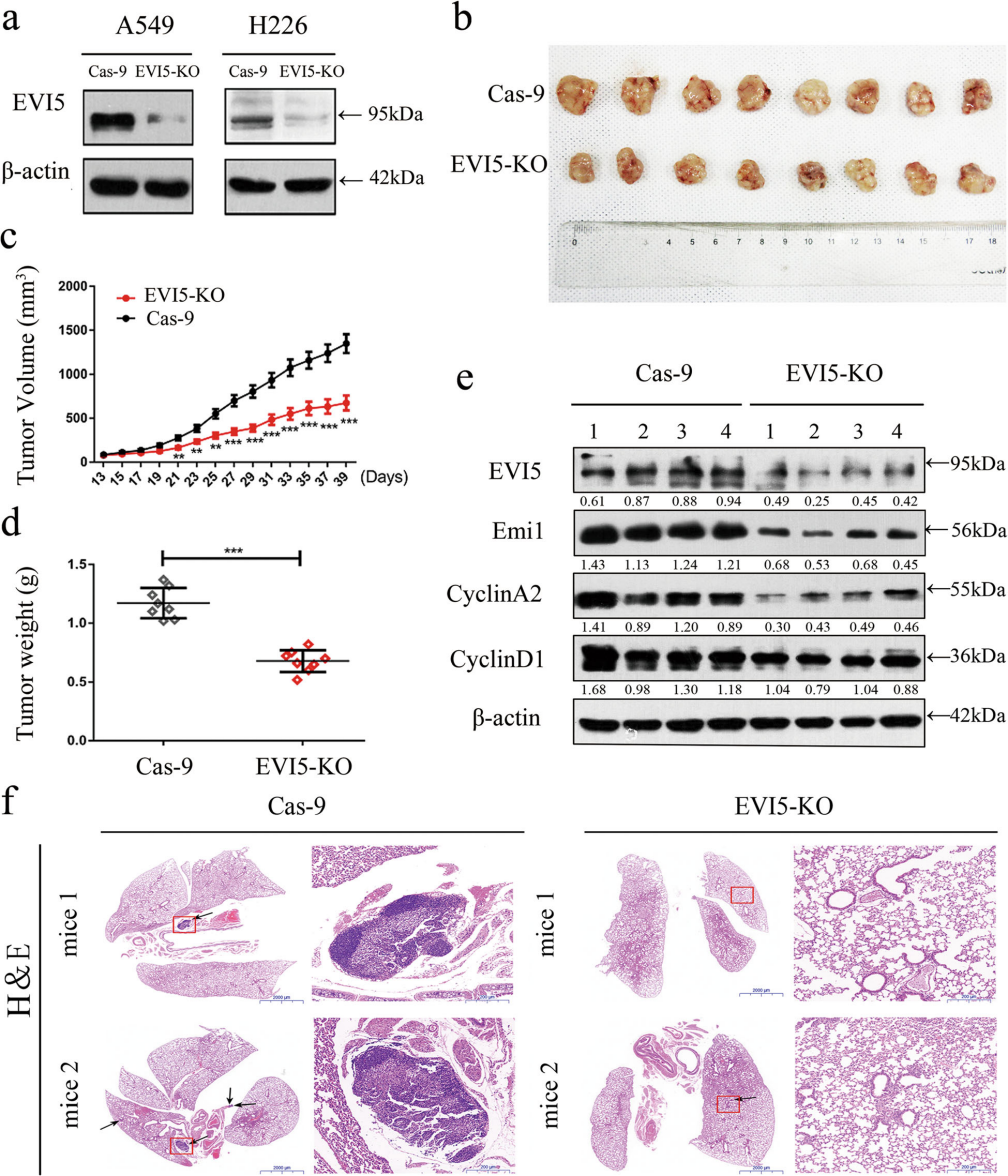
Inhibition of in vivo tumor growth and lung metastasis by EVI5 knockout. **a** EVI5 protein levels in EVI5-KO NSCLC cells. **b-c** EVI5-KO A549 cell xenografts in nude mice (*n* = 8) at the experimental endpoint; tumours were harvested and photographed as shown. Tumour growth curves in mice (*n* = 8 in each group) inoculated with the indicated cells at the indicated days. **d** Each tumour formed was weighed. **e** EVI5 protein expression in tumours was measured by western blot analysis. **f** H&E staining confirmed the presence of tumour cells in the indicated lung sections. The data are shown as the mean ± SD, Significant differences compared with the control: ***P < 0.001. Abbreviations: Cas-9, Lenti-CRISPR v2; EVI5-KO, EVI5 knockout

To clarify the cellular mechanism underlying EVI5-mediated tumor progression, we established stable EVI5-knockout NSCLC cell lines (EVI5-KO cells), this was confirmed by the barely expressed level of EVI5 protein expression in EVI5-KO cells compared to that in vector transfected cells (Cas-9 cells) (Fig. 3a). The results of phenotypic experiments in stable EVI5-KO cell lines strongly suggested that EVI5 functions as a oncogene in NSCLC (Additional file 4: Figure S3). Control A549 cells and the corresponding stable EVI5-KO A549 cells were inoculated into BALB/C athymic mice. Tumors formed by the cells with EVI5 knockout were much smaller in size than those formed from the control cells (Fig. 3b, c). In line with these results, tumor weight was found to be lower in cells with EVI5 knockout (Fig. 3d). The tissues resected from the xenograft tumors were analyzed to verify EVI5 expression using western-blot (Fig. 3e). Further, Hematoxylin and esosin (H&E) staining was used to analyze the lung metastasis between two groups, a total of 5 mice in the Cas-9 group had lung metastasis, while the EVI5-KO group had lung metastasis merely by one mouse. There were 9 metastatic foci in Cas-9 group and 1 metastatic foci in EVI5-KO group, the typical results are presented in Fig. 3f.

### EVI5 regulates the expression of cell cycle proteins by accumulating Emi1 in NSCLC

**Fig. 4:**
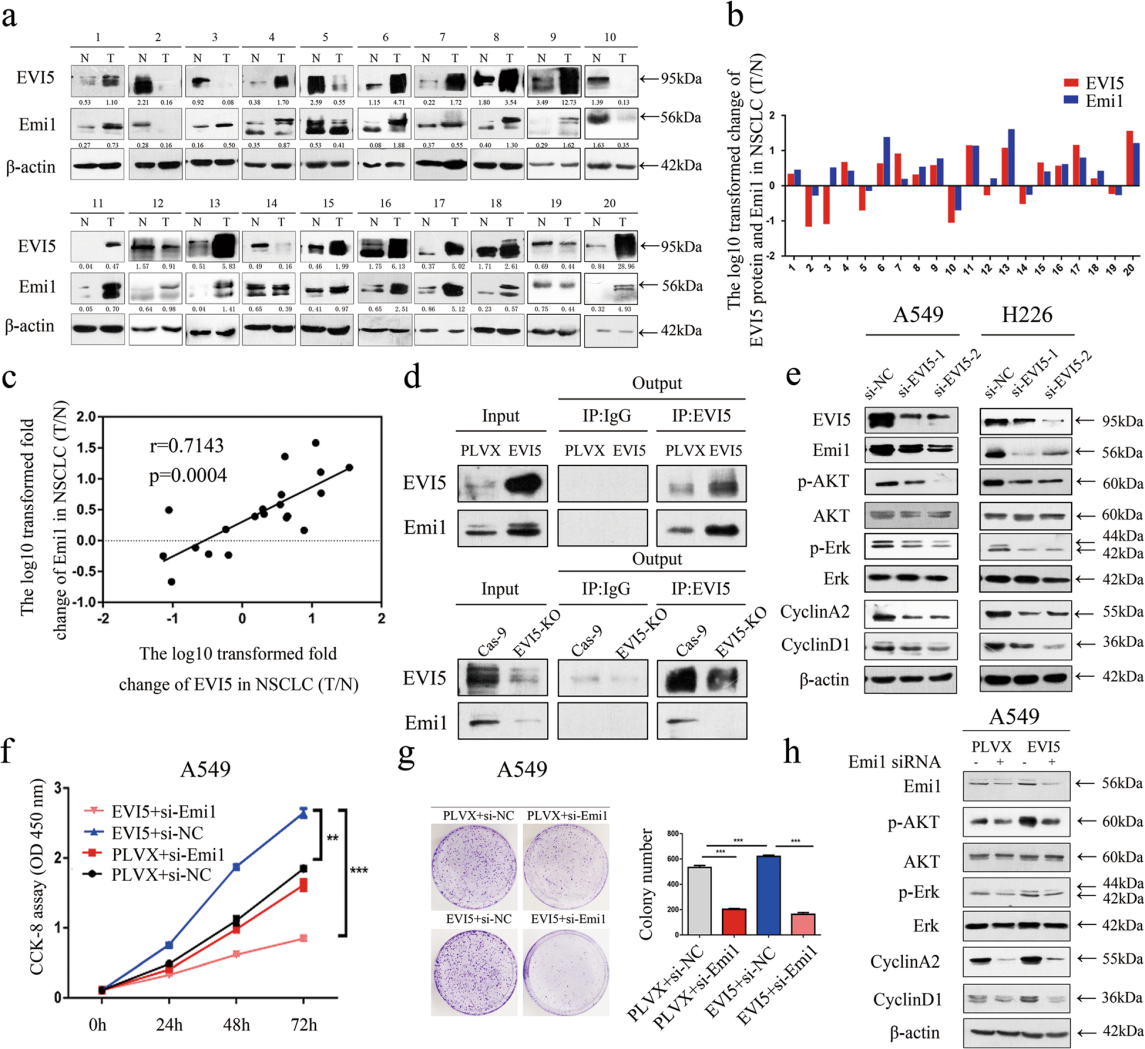
EVI5 affects the proliferation of NSCLC cells by regulating the accumulation of Emi1. **a** Western blot analysis of EVI5 and Emi1 protein levels in 20 randomly selected NSCLC tissues and paired noncancerous lung tissues. The band density ratios represent the relative expression levels of EVI5 and Emi1. **b** Relative protein expression levels of EVI5 and Emi1 in 20 paired NSCLC tissues. The Y axis indicates the log10 transformed fold change in the T/N protein expression ratios of EVI5 and Emi1. The number of each specimen is indicated below the X axis. **c** Correlation between EVI5 and Emi1 protein expression in 20 paired NSCLC tissues. The X and Y axes indicate the log10 transformed fold change in the T/N protein expression ratios of EVI5 and Emi1, respectively (*P* < 0.001). **d** Co-immunoprecipitation of EVI5 and Emi1 are shown. Protein were immunoprecipitated and detected from lysates of control and EVI5-overexpressing or EVI5-KO A549 cells using a specific monoclonal antibody. **e** The levels of Emi1 and downstream signaling molecules were measured; the levels of Emi1, p-Akt, p-Erk, CyclinA2, and CyclinD1 were significantly decreased in EVI5-knockdown cells compared with control cells. **f** CCK-8 assay to assess the viability of EVI5-overexpressing A549 cells transfected with siRNAs targeting Emi1 or with si-NC. Cell viability was assessed at 24, 48 and 72 h. **g** Representative images of the clonogenic assay results for cell proliferation in EVI5-overexpressing A549 cells transfected with siRNAs targeting Emi1 or with si-NC. Bar charts showing the clonogenic growth of cells. **h** EVI5-overexpressing A549 cells were transfected with siRNAs targeting Emi1 or with si-NC, and the expression of various proteins was then measured by western blot analysis. β-actin was used as the internal control. Bars represent mean ± SD from three independent experiments. Significant differences compared with the control: ** *P* < 0.01; ***P < 0.001. Abbreviations: PLVX, PLVX-IRES-Neo vector

Based on the findings reported in the literature [5, 10], we hypothesized that dysregulation of EVI5 could affect the cell cycle of NSCLC by causing the accumulation of Emi1. And we verified that there was a certain correlation between EVI5 and Emi1 in NSCLC in vivo. To verify the relationship between EVI5 and Emi1 in NSCLC carcinogenesis, we re-randomized 20 paired NSCLC tissues and adjacent noncancerous lung tissues. The clinicopathological parameters of these 20 paired samples are shown in Additional file 5: Table S2. As illustrated in Fig. 4a, b, there were 13 cases of NSCLC with higher level of EVI5 protein and 15 cases of NSCLC tissue with higher level of Emi1 protein in 20 paired NSCLC tissues. Interestingly, there was a positive correlation between EVI5 protein level (T/N) and Emi1 protein level (T/N) (Fig. 4c). Combined with the previous findings, we examined complexes of EVI5 and Emi1 using co-immunoprecipitation, the result showed that EVI5 overexpression in NSCLC indeed significantly increased expression of Emi1, and EVI5 knockout in NSCLC indeed significantly decreased expression of Emi1 (Fig. 4d). As illustrated in Fig. 4e, our data showed that the Emi1, p-Akt and p-Erk, CyclinA2 and CyclinD1 levels were significantly decreased in the EVI5-knockdown cells compared with the control cells. Furthermore, in cell lines with EVI5 overexpression, knockdown of Emi1 induced decrease the proliferation ability induced by EVI5 overexpressing (Fig. 4f, g) and reduced the levels of p-Akt and its associated downstream signaling molecules induced by EVI5 overexpressing (Fig. 4h).

### EVI5 promotes cell migration and invasion by regulating TGF-β1 induced signaling pathway in NSCLC

**Fig. 5:**
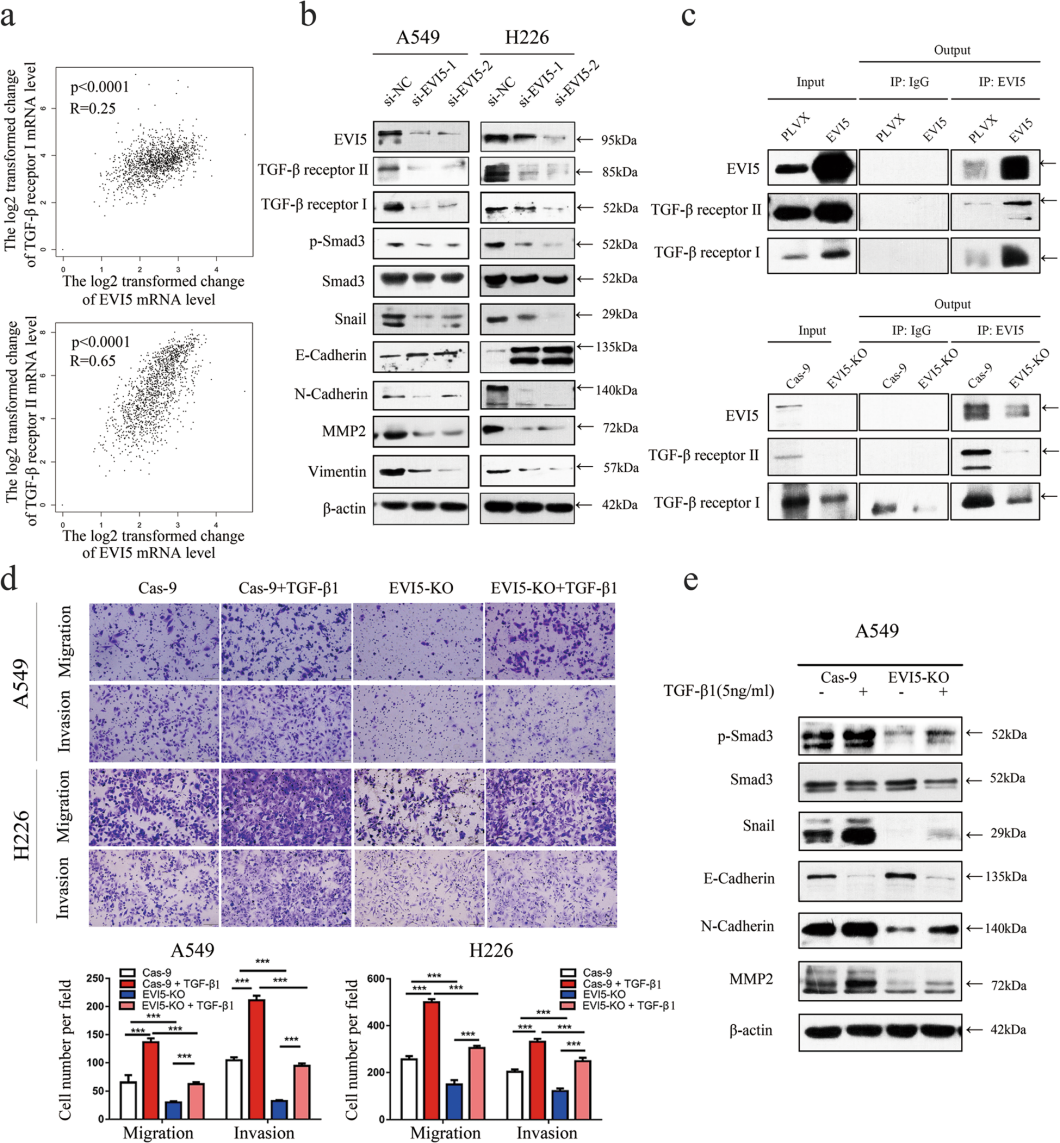
EVI5 mediates the migration and invasion of NSCLC cells through the TGF-β/Smad signaling pathway. **a** Data obtained from several study groups deposited in the GEPIA2 database were analysed to explore the correlation between EVI5 and TGF-β receptors mRNA levels. **b** The levels of TGF-β receptor II, TGF-β receptor I, p-Smad3, Snail, N-cadherin, and MMP2 were significantly decreased and E-Cadherin was significantly increased in EVI5-knockdown cells compared with control cells. **c** Co-immunoprecipitation of EVI5 and TGF-β receptor II were shown, protein were immunoprecipitated from lysates of control and EVI5-overexpressing or EVI5-KO A549 cells using a specific monoclonal antibody. **d** EVI5-KO A549 and H226 cells treated or untreated with TGF-β1 (5 ng/ml) were allowed to migrate through 8-mm pore size transwell inserts. One day later, migratory and invasive cells were stained and counted in at least three light microscopic field, One representative image is shown (Cas-9 group compared with EVI5-KO group, Cas-9 + TGF-β1 group compared with EVI5-KO + TGF-β1 group). **e** EVI5-KO A549 cells were treated with TGF-β1 (5 ng/ml) for 24 h, and the expression levels of various proteins were then measured by western blot analysis. β-actin was used as the internal control. Bars represent mean ± SD from three independent experiments. Significant differences compared with the control:***P < 0.001

Based on GEPIA2 database (http://gepia.cancer-pku.cn/), we found that the EVI5 mRNA was positively correlated with TGF-β receptors mRNA level in NSCLC, especially TGF-β receptor II (Fig. 5a). To clarify the mechanism underlying EVI5-mediated tumor metastasis, we analyzed the molecular expression of TGF-β receptor II, TGF-β receptor I, p-Smad3, Snail, Vimentin, MMP2 and N-Cadherin levels were significantly decreased and E-Cadherin was significantly increased in EVI5-knockdown cells (Fig. 5b). Next, we examined complexes of EVI5 and TGF-β receptors using co-immunoprecipitation, EVI5 overexpression in NSCLC indeed significantly increased expression of TGF-β receptors, and EVI5 knockout in NSCLC indeed significantly decreased expression of TGF-β receptors (Fig. 5c). Then, we found that the migration and invasion abilities of NSCLC cell lines induced by exogenous TGF-β1 were significantly inhibited by EVI5 knockout (Fig. 5d). We also found that the protein level of p-Smad3, Snail, MMP2, N-Cadherin induced by TGF-β1 were significantly inhibited by EVI5 knockout, E-Cadherin inhibited by TGF-β1 was significantly promoted by EVI5 knockout (Fig. 5e).

### MiR-486-5p decreased cell proliferation, migration and invasion abilities of NSCLC cells by suppressing EVI5 and its downstream signaling pathway

**Fig. 6:**
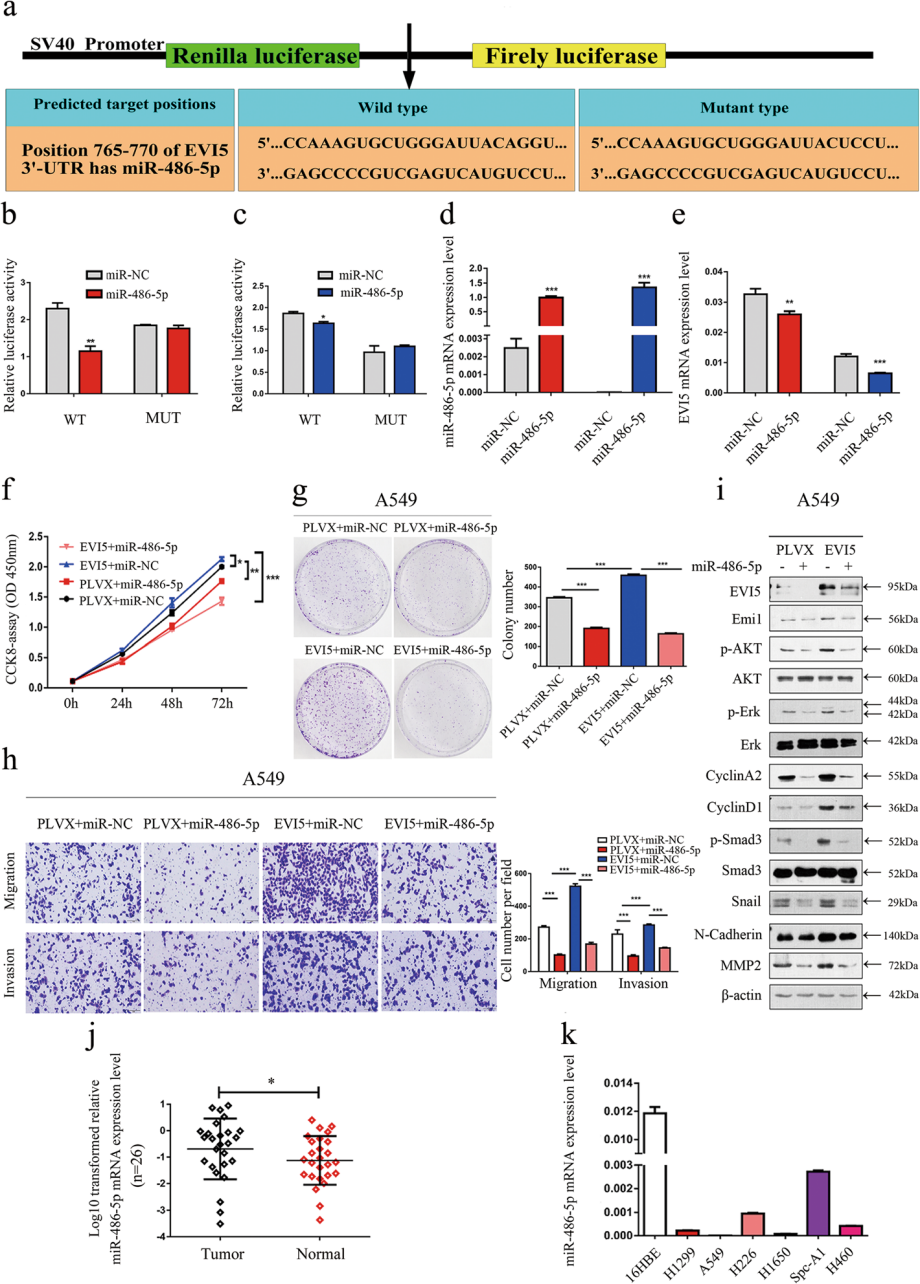
MiR-486-5p suppresses EVI5 expression and reduces the proliferation, migration and invasion capability of NSCLC cells. **a** Schematic diagram showing the subcloning of a fragment containing the predicted miR-486-5p-binding site at positions 765–770 in the EVI5 3′-UTR into the psiCHECK-2 luciferase vector. Predicted duplex formation between miR-486-5p and the wild-type or mutant miR-486-5p-binding site is indicated. **b-c** Luciferase activity of the constructs containing the wild-type or the mutant EVI5 reporter gene in A549 and H226 cells co-transfected with miR-NC or miR-486-5p. A scrambled sequence was used as the NC. The relative Renilla luciferase activity was measured and normalized to firefly luciferase activity. **d-e** The expression of miR-486-5p and EVI5 in NSCLC cells transfected with miR-486-5p mimics was measured by qRT-PCR. **f** CCK-8 assay of cell viability in EVI5-overexpressing A549 cells transfected with miR-486-5p mimics or miR-NC. **g** Representative images of the clonogenic assay results for cell proliferation in EVI5-overexpressing A549 cells transfected with miR-486-5p mimics or miR-NC. **h** Representative images of the transwell assay results for cell migration and invasion in EVI5-overexpressing A549 cells transfected with miR-486-5p mimics or miR-NC. **i** EVI5-overexpressing A549 cells were transfected with miR-486-5p or miR-NC, and the expression levels of various proteins were then measured by western blot analysis. **j** The mRNA expression levels of miR-486-5p were measured by qRT-PCR and compared between 26 NSCLC and paired adjacent noncancerous lung tissues. **k** qRT-PCR analysis of relative miR-486-5p expression levels in human NSCLC cell lines. β-actin was used as the internal control. Bars represent mean ± SD from three independent experiments. Significant differences compared with the control: * *P* < 0.05; ** *P* < 0.01; ***P < 0.001

Ample evidence showed that EVI5 expression was elevated in human cancers [24, 25], while the underlying mechanisms are poorly understood. Herefore we focused on NSCLC cells to investigate whether miRNAs can epigenetically influence EVI5 expression. In silico prediction (microRNA.org) showed that EVI5 is a potential target of miR-486-5p. miR-486-5p possibly inhibits EVI5 expression by directly binding to its 3′-UTR region (Fig. 6a). To verify this, a dual-luciferase reporter vector containing the EVI5 3′-UTR seed region specific to miR-486-5p, or the corresponding mutant sequence was used. As shown in Fig. 6b and c, the luciferase activities of the reporter vector containing the seed region of miR-486-5p in NSCLC cells was significantly suppressed by miR-486-5p transfection. The data showed that the expression of miR-486-5p was increased in NSCLC cells transfected with the miR-486-5p mimics (Fig. 6d). In correspondence with the expression of miR-486-5p, the level of EVI5 was downregulated in cells transfected with miR-486-5p mimics (Fig. 6e). Then, we found that in EVI5 overexpression cells, transfected with miR-486-5p mimics decreased the proliferation, migration and invasion, less than in control cells transfected with miR-486-5p mimics (Fig. 6f-h). And in EVI5 overexpression cells, transfected with miR-486-5p mimics decreased the protein level of Emi1, p-Akt, p-Erk, CyclinA2, CyclinD1, TGF-β receptor II, TGF-β receptor I, p-Smad3, Snail, MMP2, N-Cadherin, its effect was still weak for control cells transfected with miR-486-5p mimics (Fig. 6i).

MiR-486-5p expression is significantly downregulated in NSCLC tissues [26]. This affects crucial cellular pathways, including cell-cell adhesion and signaling transduction [27], cell cycle regulation and apoptosis [28], and plays a significant role in the pathogenesis of NSCLC [29]. Then, we used qRT-PCR assay to detect the mRNA expression of miR-486-5p in NSCLC tissues, due to missing samples, 26 (65%) of the 40 paired NSCLC tissues and adjacent noncancerous lung tissues mentioned above were used, and we found that its expression was significantly reduced in tumor tissues compared with the paired noncancerous tissues from the same patient (Fig. 6j and Additional file 6: Table S3). And in these 26 paired tissues, a total of 14 (54%) EVI5 expression levels were negatively correlated with miR-486-5p expression levels (Additional file 7: Figure S4). Consistent with the data above, miR-486-5p was also downregulated in several NSCLC cell lines compared with its level in 16HBE cells (Fig. 6k).

### MiR-486-5p overexpression decreased cell proliferation, migration and invasion abilities in vitro

**Fig. 7:**
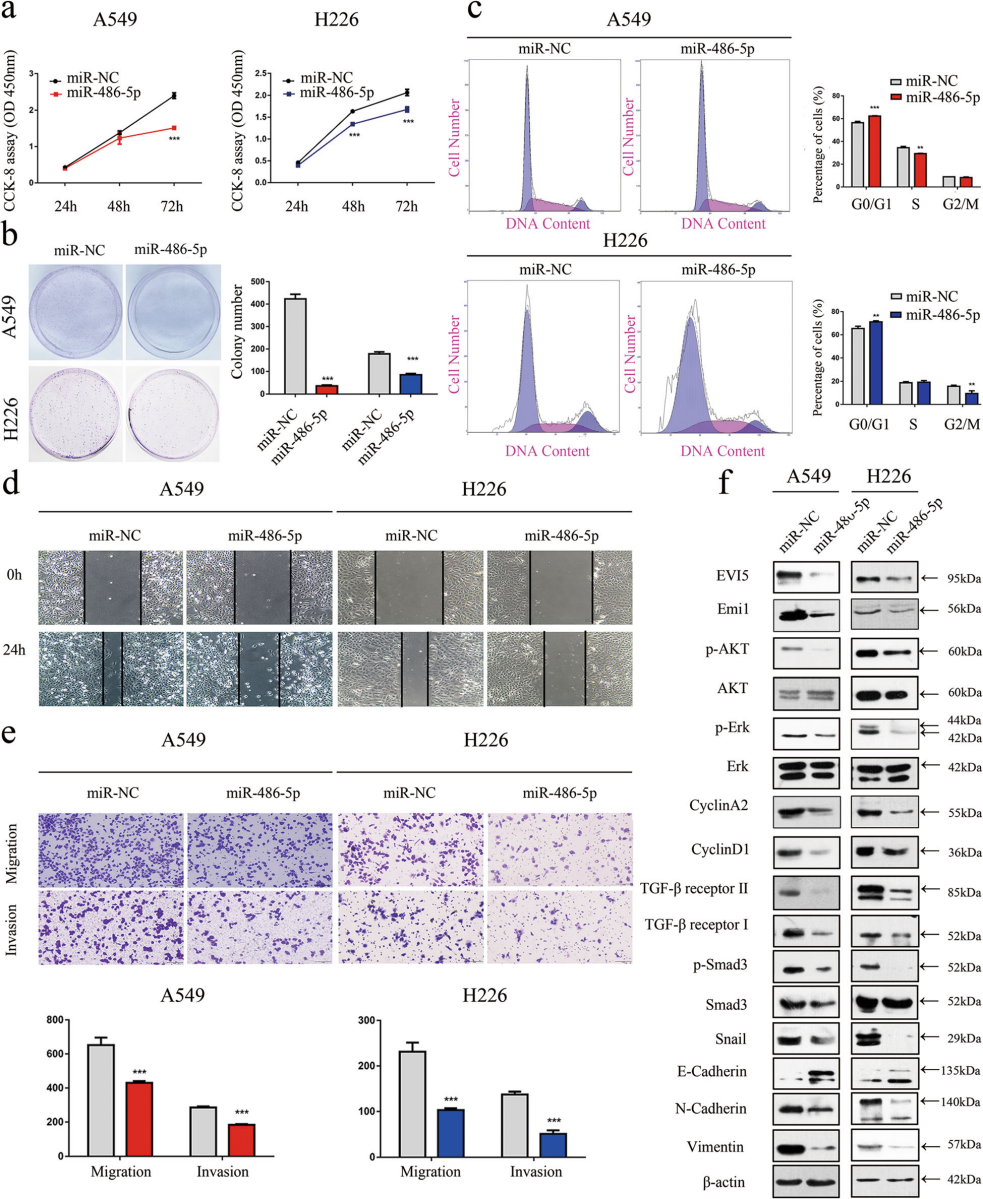
Inhibition of NSCLC cell proliferation, migration and invasion by overexpressing miR-486-5p. **a** CCK-8 assay of cell viability in NSCLC cell lines transfected with miR-486-5p mimics at 24, 48, and 72 h. **b** Representative images of the clonogenic assay results for NSCLC cell proliferation. **c** Flow cytometric analysis of A549 and H226 cells (cells transfected with miR-486-5p mimics vs. miR-NC). Cells were harvested 72 h after transfection and stained with Annexin V/FITC and PI. **d** Wound healing assay was performed to evaluate the effect of miR-486-5p transfection on cells. **e** Representative images of the transwell assay results for cell migration and invasion in A549 and H226 cells transfected with miR-486-5p mimics or miR-NC. **f** A549 and H226 cells were treated with or without miR-486-5p mimics for 72 h. The levels of Emi1, p-Akt, Akt, p-Erk, Erk, CyclinA2, Cyclin D1, TGF-β receptor II, TGF-β receptor I, p-Smad3, Snail, E-cadherin and N-cadherin were analysed by western blotting. β-actin was used as the internal control. Bars represent mean ± SD from three independent experiments. Significant differences compared with the control: ** *P* < 0.01; ***P < 0.001

To confirm the role of miR-486-5p in NSCLC, we transfected NSCLC cells transiently with miR-486-5p mimics or the miR-NC, the results showed that NSCLC cells overexpressing miR-486-5p had significantly lower proliferation (Fig. 7a, b). To determine how miR-486-5p suppressed cell proliferation in NSCLC cells, we tested the distribution of cell cycle phases in A549 and H226 cells, and we found that the proportion of cells in the G0/G1 phase were significantly higher, meanwhile the proportion of cells in the S phase was significantly lower in A549 cells with overexpressed miR-486-5p (Fig. 7c). In the wound healing assay, NSCLC cells overexpressed with miR-486-5p migrated towards to the scratch more slowly than did the control cells (Fig. 7d). Transwell assay of the NSCLC stable cells lines further indicated that overexpression of miR-486-5p considerably suppressed the migration and invasion abilities of NSCLC cells (Fig. 7e). Then, the protein level of EVI5 and its downstream signaling molecules were determined between the cells overexpressing miR-486-5p or miR-NC. In line with the results for EVI5-knockdown cells, our data showed that overexpression of miR-486-5p significantly suppressed the protein levels of EVI5, Emi1, p-Akt, p-Erk, CyclinA2, and CyclinD1 (Fig. 7f), as well as the upregulation of E-cadherin and downregulation of TGF-β receptor II, TGF-β receptor I, p-Smad3, N-cadherin, Vimentin, Snail levels (Fig. 7f).

## Discussion

Malignant tumor is essentially a disease involving unlimited cell proliferation [30] and metastasis [31]. In recent years, EVI5, an important protein regulating cell cycle [32] and migration [20], has been increasingly reported to be associated with HCC [24], bladder cancer [33], melanoma [34], leukemia [35], lymphoma [25] and many other tumors. Here, we identified EVI5 as a novel prognostic biomarker for NSCLC.

In the present study, we first analyzed the expression of EVI5 in NSCLC tissues, its expression was upregulated in NSCLC tissues compared with that of matched paracancerous tissues. Moreover, we identified the higher expression of EVI5 in the NSCLC cell lines, which shows that EVI5 is frequently overexpressed in NSCLC. Our findings indicated that EVI5 significantly promotes NSCLC cell proliferation by accelerating the NSCLC cell cycle. The co-expression of EVI5 and Emi1 has been reported in other types of cancers [10]. Consistent with the previous findings, EVI5 was found to promote Emi1 and Cyclins expression in our study. Therefore, our findings demonstrated that EVI5 may play a protumoral role in NSCLC via it effects on Emi1 accumulation. However, the interactions and underlying mechanisms need to be elucidated.

The public dataset from Kaplan-Meier Plotter indicated that higher expression of EVI5 was significantly associated with poor survival of patients with NSCLC. Metastasis is a critical issue leading to poor survival, as lymph node metastasis is a fundamental factor in the determination of the clinical staging and prognosis of NSCLC. The TGF-β/Smad signaling pathway plays an important role in EMT progression in various epithelial cell types [36] and thus, ongoing research has focused on investigating ways to reverse or delay the EMT to prevent carcinogenic progression. Furthermore, as a GTPase that regulates intracellular transport, membrane trafficking, cytokinesis, and cell migration [37], the tumor promoting metastasis role of EVI5 is poorly understood. When EVI5 knockdown in NSCLC, the TGF-β receptor II, TGF-β receptor I, p-Smad3, Snail, Vimentin, MMP2 and N-Cadherin levels were significantly decreased and E-Cadherin was significantly increased. The administration of TGF-β1 increased the migration and invasion of EVI5-knockout NSCLC cells, and increased the levels of p-Smad3 and its downstream signaling molecules, indicating a therapeutic role for EVI5 in inhibition of cancer metastasis. In the present study, we have reported for the first time that EVI5 could promote TGF-β/Smad induced cell migration and invasion by interacting with TGF-β receptors in NSCLC. Next, We sought to investigate the in-depth mechanism in which EVI5 may involved to be a binding partner and GTPase-activating protein domain for Rab11, and may influence the ability of Rab11 to recycling of endocytosed TGF-β receptors to the plasma membrane [21]. This requires further exploration to reveal how EVI5 interacts with TGF-β receptors, thus affecting the metastasis of NSCLC.

As an important gene, EVI5 has only been reported to be regulated by miR-135b in HCC [38]. Although there is ample evidence for the upregulation of EVI5 expression in NSCLC, the underlying mechanisms are poorly understood. The malignant progression of NSCLC is considered to be a comprehensive event that includes a gene expression network and alterations in the tumor microenvironment, in which microRNAs play critical roles [39]. MiR-486-5p, widely documented to be a tumor-suppressive microRNA, inhibits proliferation and invasion in many types of cancers [39, 40], In the present study, we observed an interaction between EVI5 and miR-486-5p. To address this question, a dual-luciferase reporter assay was performed, and it showed that miR-486-5p significantly inhibited luciferase activity in cells transfected with the wild-type EVI5 3′-UTR, Moreover, overexpression of miR-486-5p prevented EVI5-induced cell proliferation, migration and invasion in NSCLC cells, which further confirms the tumor-promoting function of EVI5 and provides more clues to the regulation network of EVI5.

## Conclusion

**Fig. 8:**
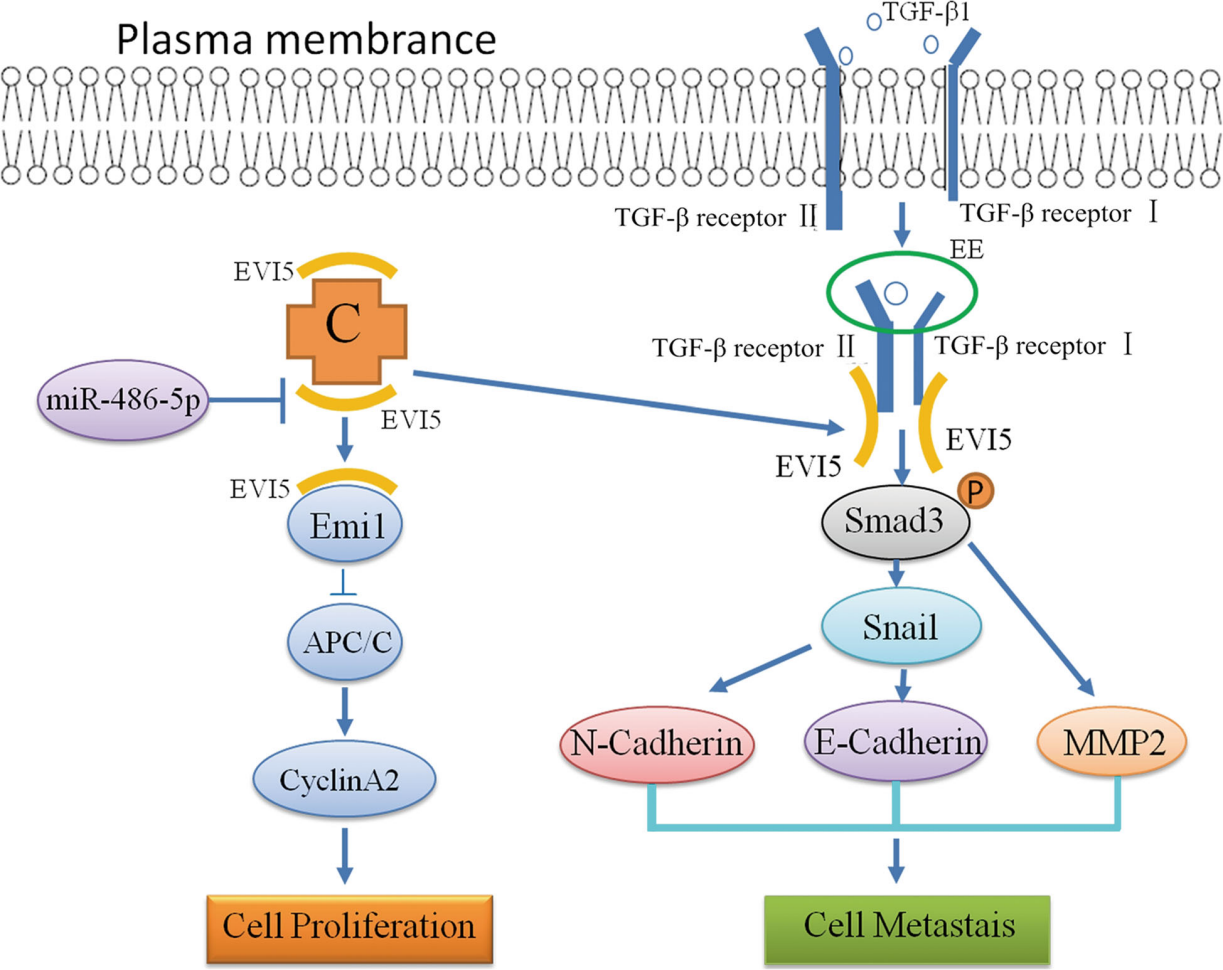
A mechanistic working model of EVI5 in controlling Emi1 accumulation or TGF- β/Smad signaling pathway: MiR-486-5p regulates EVI5 expression via Emi1 or TGF-β/Smad signaling in NSCLC

Taken together, our study is the first to report that EVI5 expression is upregulated in NSCLC and is negatively correlated with miR-486-5p expression. Further, we found that EVI5 affects the efficacy of Emi1-targeted cell cycle dysregulation in NSCLC cells. In addition, EVI5 was shown to activate downstream Smad3 signaling pathways by interacting with TGF-β receptors, We integrated these findings in a model showing the pivotal role of EVI5 in the regulation in NSCLC (Fig. 8). Our study provides new insights into the theoretical basis of NSCLC and therapeutic strategies for its treatment.

## Supplementary information

**Additional file 1: Table S1.** Demographic and clinical characteristics and levels of EVI5 mRNA expression in NSCLC tissue.

**Additional file 2: Figure S1.** Inhibition of NSCLC cell proliferation and apoptosis by EVI5 knockdown. **a** The clonogenic assay of A549 and H226 cells (si-EVI5 compared with si-NC). **b** The flow cytometry results indicated that transfection of NSCLC cells with si-EVI5 resulted in an increase in apoptosis. Bars represent mean ± SD from three independent experiments. Significant differences compared with the control: **P < 0.01; ***P < 0.001.

**Additional file 3: Figure S2.** Promotion of NSCLC cell pathogenesis by EVI5 overexpression. **a** EVI5 mRNA and protein levels in EVI5-overexpressing NSCLC cells. **b** CCK-8 assay of cell viability in EVI5-overexpressing A549 cells; cell viability was assessed at 24, 48 and 72 h. **c** Representative images of the clonogenic assay results for cell proliferation in EVI5-overexpressing A549 cells. **d** Representative images of the transwell assay results for cell migration and invasion in A549 and H226 cells (EVI5 overexpressing compared with PLVX). β-actin was used as the internal control. Bars represent mean ± SD from three independent experiments. Significant differences compared with the control: ***P < 0.001.

**Additional file 4: Figure S3.** Inhibitory effect of EVI5 knockout on the pathogenesis of NSCLC cells. **a** CCK-8 assay of cell viability in A549 and H226 cells (EVI5-KO groups compared with Cas-9 groups). **b** Representative images of the transwell assay results for cell migration and invasion in A549 and H226 cells (EVI5-KO groups compared with Cas-9 groups). Bars represent mean ± SD from three independent experiments. Significant differences compared with the control: * *P* < 0.05; ***P < 0.001.

**Additional file 5: Table S2.** Demographic and clinical characteristics and levels of EVI5 protein expression in NSCLC tissue.

**Additional file 6: Table S3.** Demographic and clinical characteristics and levels of miR-486-5p mRNA expression in NSCLC tissue.

**Additional file 7: Figure S4.** Relative mRNA expression levels of EVI5 and miR-486-5p in 26 paired NSCLC tissues. The Y axis indicates the log10 transformed fold change in the T/N mRNA expression ratios of EVI5 and miR-486-5p. The number of each specimen is indicated below the X axis.

## Data Availability

The datasets used and/or analyzed during the current study are available from the corresponding author on reasonable request.

## Abbreviations

EVI5: Ecotropic viral integration site 5
APC/C: Anaphase-promoting complex/cyclosome
NSCLC: Non-small cell lung cancer
GAPs: GTPase-activating proteins
TGF-β1: Transforming growth factor-β1
EMT: Epithelial–mesenchymal transition
microRNA: miRNA
qRT-PCR: Quantitative reverse transcription–PCR
Cas-9: Lenti-CRISPR v2
PLVX: PLVX-IRES-Neo vector
EVI5-KO: EVI5 knockout
