# Supplementary material for: EVI5 is an oncogene that regulates the proliferation and metastasis of NSCLC cells
Source: J Exp Clin Cancer Res. 2020 May 11;39:84. doi: 10.1186/s13046-020-01585-z (PMC7212589; doi:10.1186/s13046-020-01585-z)
Supplement: Supplementary file 1 — Additional file 1: Table S1. Demographic and clinical characteristics and levels of EVI5 mRNA expression in NSCLC tissue. [file 13046_2020_1585_MOESM1_ESM.doc]

­­

Addition flie 1: Table S1. Demographic and clinical characteristics and levels of EVI5 mRNA expression in NSCLC tissue

| Characteristics | n=40 | EVI5 mRNA expression  high(n=24) low(n=16) | | *P* value |
| --- | --- | --- | --- | --- |
| Age (years) |  |  |  |  |
| ≤60 | 14(35.0%) | 8 | 6 | 0.787 |
| >60 | 26(65.0%) | 16 | 10 |
| Gender |  |  |  |  |
| Male | 22(55.0%) | 13 | 9 | 0.897 |
| Female | 18(45.0%) | 11 | 7 |
| Histological features |  |  |  |  |
| Adenocarcinoma | 27(65.0%) | 18 | 8 | 0.437 |
| Squamous cell carcinoma | 8(20.0%) | 4 | 4 |
| Others | 6(15.0%) | 2 | 4 |  |
| Degree of differentiation |  |  |  |  |
| Low | 10(25.0%) | 5 | 5 | 0.456 |
| Middle | 30(75.0%) | 19 | 11 |  |
| Smoker |  |  |  |  |
| Yes | 12(30.0%) | 7 | 5 | 0.888 |
| No | 28(70.0%) | 17 | 11 |
| Clinical stage |  |  |  |  |
| I +II | 24(60.0%) | 17 | 7 | 0.087 |
| III + IV | 16(40%) | 7 | 9 |
| Distant metastasis |  |  |  |  |
| No | 35(87.5%) | 23 | 12 | 0.051 |
| Yes | 5(12.5%) | 1 | 4 |  |
| Lymph node metastasis |  |  |  |  |
| No | 21(52.5%) | 12 | 9 | 0.698 |
| Yes | 19(47.5%) | 12 | 7 |

Data are presented as mean ± SD values. Kruskal-Wallis test for comparison between three or more groups.
