# Supplementary figures and images for: EVI5 is an oncogene that regulates the proliferation and metastasis of NSCLC cells
Source: J Exp Clin Cancer Res. 2020 May 11;39:84. doi: 10.1186/s13046-020-01585-z (PMC7212589; doi:10.1186/s13046-020-01585-z)

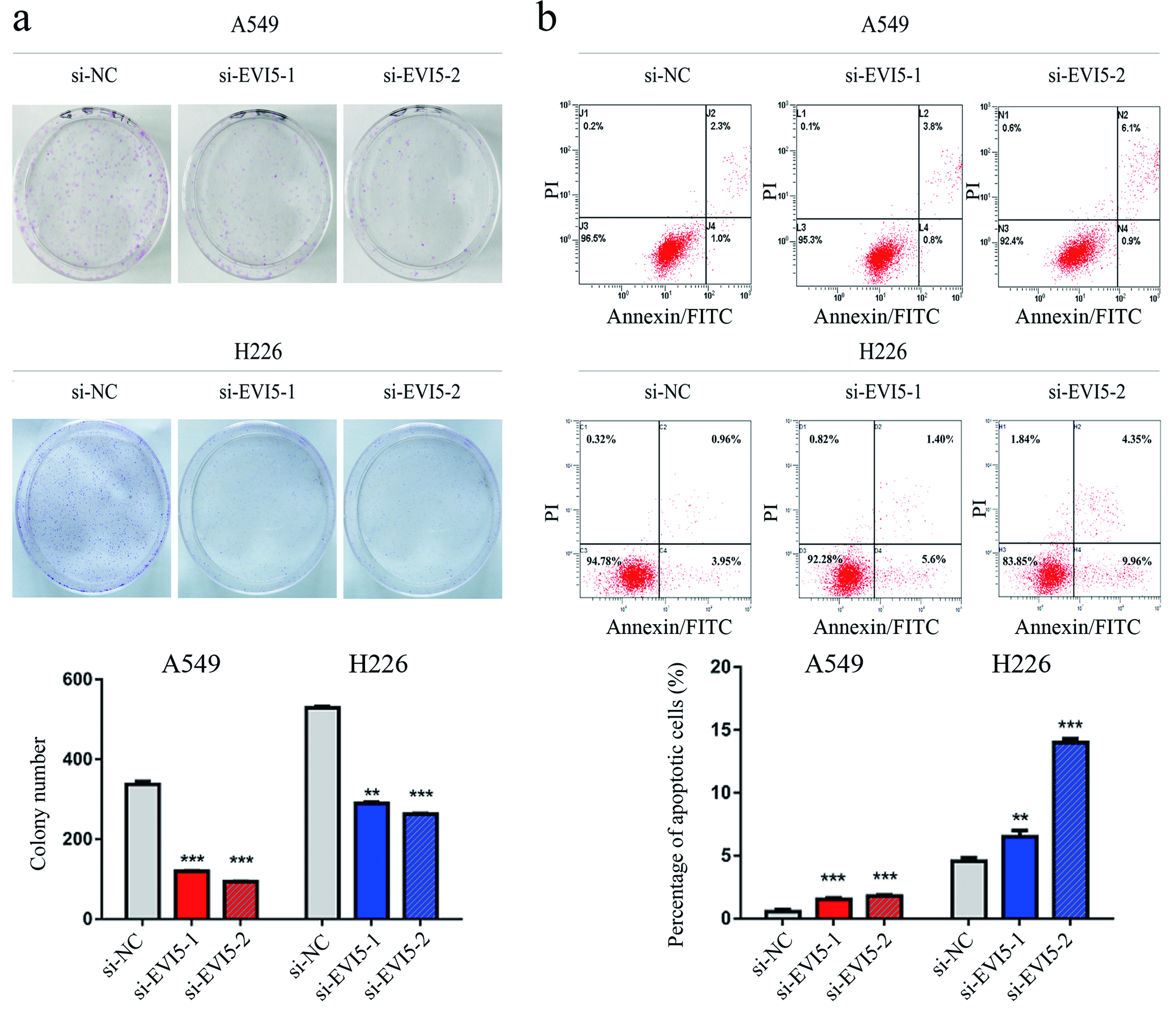

Supplement: Supplementary file 2 — Additional file 2: Figure S1. Inhibition of NSCLC cell proliferation and apoptosis by EVI5 knockdown. a The clonogenic assay of A549 and H226 cells (si-EVI5 compared with si-NC). b The flow cytometry results indicated that transfection of NSCLC cells with si-EVI5 resulted in an increase in apoptosis. Bars represent mean ± SD from three independent experiments. Significant differences compared with the control: **P < 0.01; ***P < 0.001. [file 13046_2020_1585_MOESM2_ESM.tif]

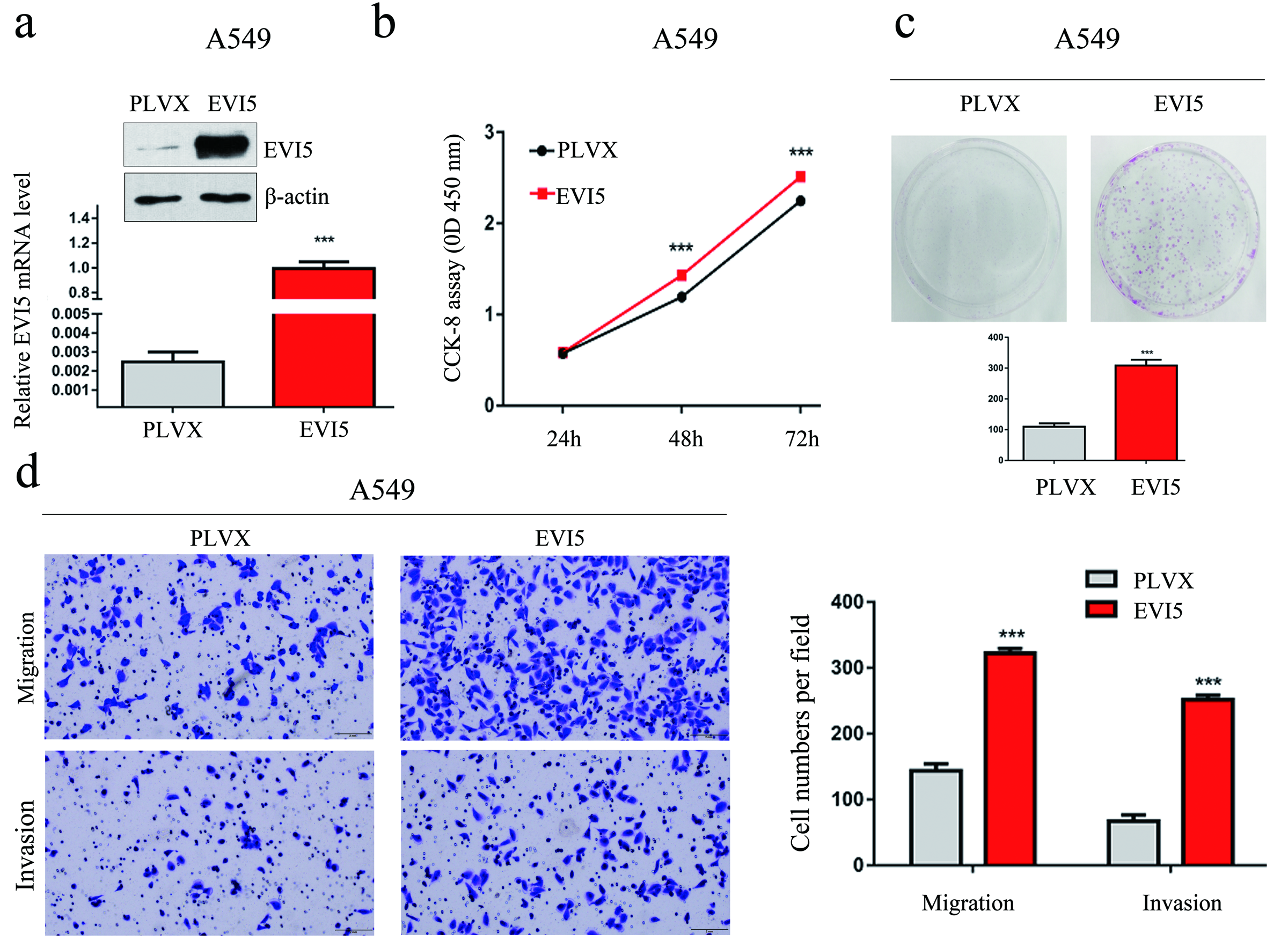

Supplement: Supplementary file 3 — Additional file 3: Figure S2. Promotion of NSCLC cell pathogenesis by EVI5 overexpression. a EVI5 mRNA and protein levels in EVI5-overexpressing NSCLC cells. b CCK-8 assay of cell viability in EVI5-overexpressing A549 cells; cell viability was assessed at 24, 48 and 72 h. c Representative images of the clonogenic assay results for cell proliferation in EVI5-overexpressing A549 cells. d Representative images of the transwell assay results for cell migration and invasion in A549 and H226 cells (EVI5 overexpressing compared with PLVX). β-actin was used as the internal control. Bars represent mean ± SD from three independent experiments. Significant differences compared with the control: ***P < 0.001. [file 13046_2020_1585_MOESM3_ESM.tif]

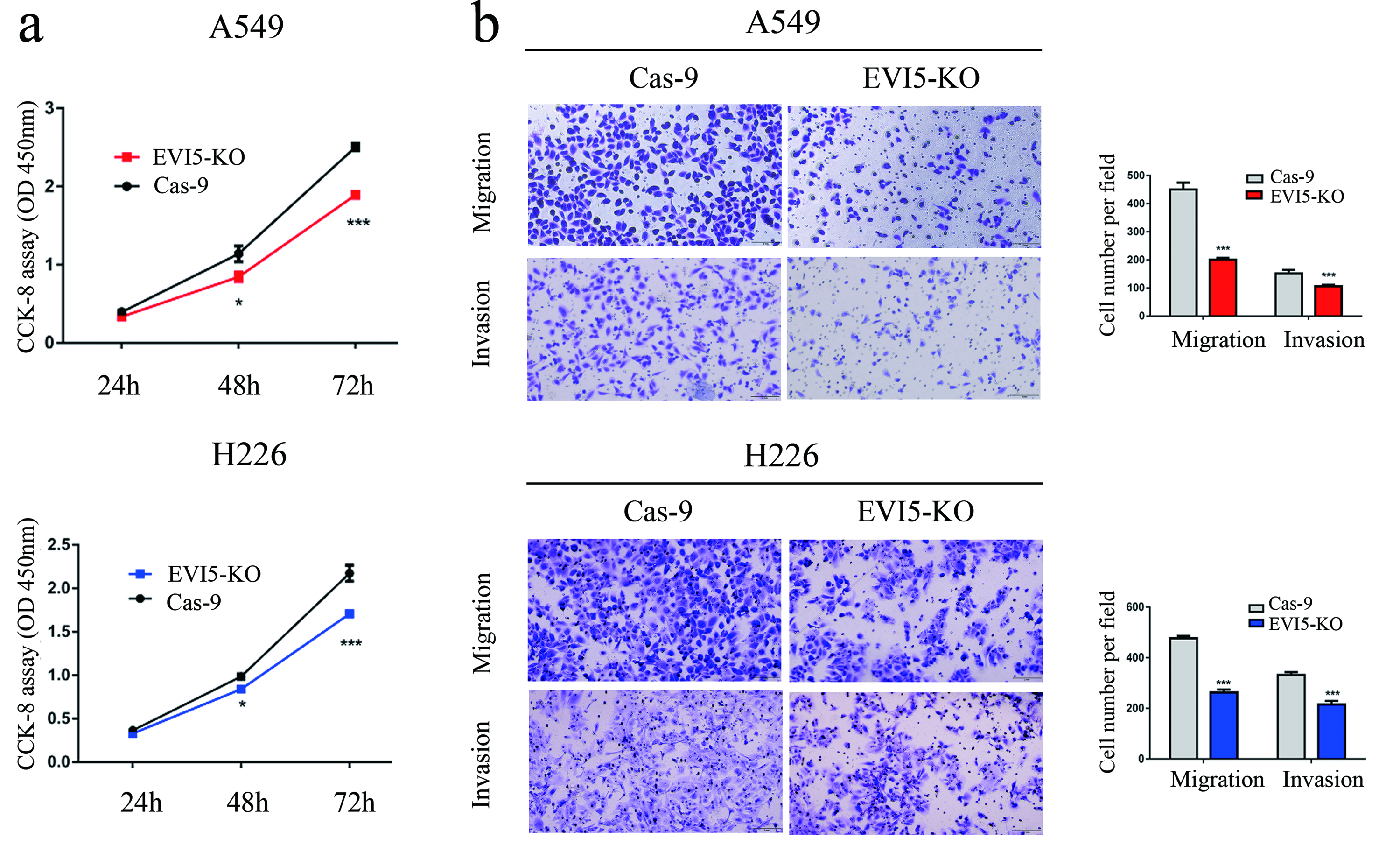

Supplement: Supplementary file 4 — Additional file 4: Figure S3. Inhibitory effect of EVI5 knockout on the pathogenesis of NSCLC cells. a CCK-8 assay of cell viability in A549 and H226 cells (EVI5-KO groups compared with Cas-9 groups). b Representative images of the transwell assay results for cell migration and invasion in A549 and H226 cells (EVI5-KO groups compared with Cas-9 groups). Bars represent mean ± SD from three independent experiments. Significant differences compared with the control: * P < 0.05; ***P < 0.001. [file 13046_2020_1585_MOESM4_ESM.tif]

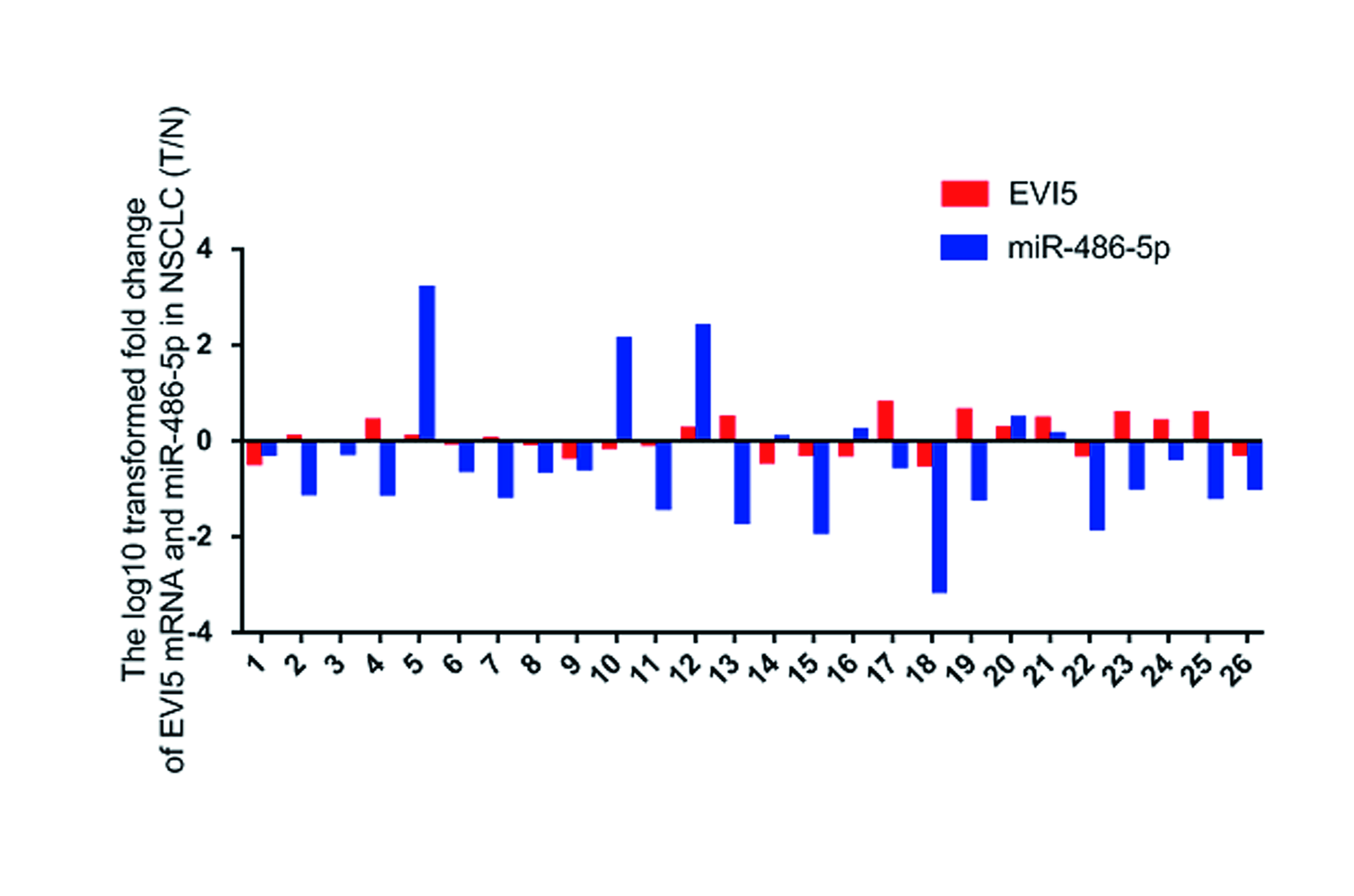

Supplement: Supplementary file 7 — Additional file 7: Figure S4. Relative mRNA expression levels of EVI5 and miR-486-5p in 26 paired NSCLC tissues. The Y axis indicates the log10 transformed fold change in the T/N mRNA expression ratios of EVI5 and miR-486-5p. The number of each specimen is indicated below the X axis. [file 13046_2020_1585_MOESM7_ESM.tif]
