# Supplementary material for: EVI5 is an oncogene that regulates the proliferation and metastasis of NSCLC cells
Source: J Exp Clin Cancer Res. 2020 May 11;39:84. doi: 10.1186/s13046-020-01585-z (PMC7212589; doi:10.1186/s13046-020-01585-z)
Supplement: Supplementary file 5 — Additional file 5: Table S2. Demographic and clinical characteristics and levels of EVI5 protein expression in NSCLC tissue. [file 13046_2020_1585_MOESM5_ESM.doc]

­­

Addition file 5: Table S2. Demographic and clinical characteristics and levels of EVI5 protein expression in NSCLC tissue

| Characteristics | n=20 | EVI5 protein expression  high(n=13) low(n=7) | | *P* value |
| --- | --- | --- | --- | --- |
| Age (years) |  |  |  |  |
| ≤60 | 10(50.0%) | 6 | 4 | 0.639 |
| >60 | 10(50.0%) | 7 | 3 |
| Gender |  |  |  |  |
| Male | 12(55.0%) | 9 | 3 | 0.251 |
| Female | 8(45.0%) | 4 | 4 |
| Histological features |  |  |  |  |
| Adenocarcinoma | 27(65.0%) | 10 | 6 | 0.517 |
| Squamous cell carcinoma | 8(20.0%) | 2 | 0 |
| Others | 6(15.0%) | 1 | 1 |  |
| Smoker |  |  |  |  |
| Yes | 12(30.0%) | 2 | 1 | 0.948 |
| No | 28(70.0%) | 11 | 6 |
| Clinical stage |  |  |  |  |
| I +II | 24(60.0%) | 10 | 7 | 0.549 |
| III + IV | 16(40%) | 3 | 1 |
| Distant metastasis |  |  |  |  |
| No | 35(87.5%) | 12 | 7 | 0.452 |
| Yes | 5(12.5%) | 1 | 0 |  |
| Lymph node metastasis |  |  |  |  |
| No | 21(52.5%) | 10 | 6 | 0.639 |
| Yes | 19(47.5%) | 3 | 1 |

Data are presented as mean ± SD values. Kruskal-Wallis test for comparison between three or more groups.
