# Supplementary material for: EVI5 is an oncogene that regulates the proliferation and metastasis of NSCLC cells
Source: J Exp Clin Cancer Res. 2020 May 11;39:84. doi: 10.1186/s13046-020-01585-z (PMC7212589; doi:10.1186/s13046-020-01585-z)
Supplement: Supplementary file 6 — Additional file 6: Table S3. Demographic and clinical characteristics and levels of miR-486-5p mRNA expression in NSCLC tissue. [file 13046_2020_1585_MOESM6_ESM.docx]

Addition file 6: Table S3. Demographic and clinical characteristics and levels of miR-486-5p mRNA expression in NSCLC tissue

| Characteristics | n=26 | miR-486-5p mRNA expression  high(n=7) low(n=19) | |
| --- | --- | --- | --- |
| Age (years) |  |  |  |
| ≤60 | 9(34.6%) | 2 | 7 |
| >60 | 17(65.4%) | 5 | 12 |
| Gender |  |  |  |
| Male | 19(73.1%) | 5 | 14 |
| Female | 7(26.9%) | 2 | 5 |
| Histological features |  |  |  |
| Adenocarcinoma | 15(57.7%) | 4 | 11 |
| Squamous cell carcinoma | 6(23.1%) | 0 | 6 |
| Others | 5(19.2%) | 3 | 2 |
| Clinical stage |  |  |  |
| I +II | 15(57.7%) | 4 | 11 |
| III + IV | 11(42.3%) | 3 | 8 |
| Distant metastasis |  |  |  |
| No | 22(84.6%) | 5 | 17 |
| Yes | 4(15.4%) | 2 | 2 |
|  |  |  |  |

Data are presented as mean ± SD values. Kruskal-Wallis test for comparison between three or more groups.
